# Nano-sized graphene oxide coated nanopillars on microgroove polymer arrays that enhance skeletal muscle cell differentiation

**DOI:** 10.1186/s40580-021-00291-6

**Published:** 2021-12-04

**Authors:** Hye Kyu Choi, Cheol-Hwi Kim, Sang Nam Lee, Tae-Hyung Kim, Byung-Keun Oh

**Affiliations:** 1 Department of Chemical and Biomolecular Engineering, Sogang University, Seoul, 04170 South Korea; 2 School of Integrative Engineering, Chung-Ang University, Seoul, 06974 Korea; 3 Uniance Gene Inc., Seoul, 04107 South Korea

**Keywords:** Nano-sized graphene oxide, Myogenesis, Micro−nano hybrid pattern, Cell behavior

## Abstract

The degeneration or loss of skeletal muscles, which can be caused by traumatic injury or disease, impacts most aspects of human activity. Among various techniques reported to regenerate skeletal muscle tissue, controlling the external cellular environment has been proven effective in guiding muscle differentiation. In this study, we report a nano-sized graphene oxide (sGO)-modified nanopillars on microgroove hybrid polymer array (NMPA) that effectively controls skeletal muscle cell differentiation. sGO-coated NMPA (sG-NMPA) were first fabricated by sequential laser interference lithography and microcontact printing methods. To compensate for the low adhesion property of polydimethylsiloxane (PDMS) used in this study, graphene oxide (GO), a proven cytophilic nanomaterial, was further modified. Among various sizes of GO, sGO (< 10 nm) was found to be the most effective not only for coating the surface of the NM structure but also for enhancing the cell adhesion and spreading on the fabricated substrates. Remarkably, owing to the micro-sized line patterns that guide cellular morphology to an elongated shape and because of the presence of sGO-modified nanostructures, mouse myoblast cells (C2C12) were efficiently differentiated into skeletal muscle cells on the hybrid patterns, based on the myosin heavy chain expression levels. Therefore, the developed sGO coated polymeric hybrid pattern arrays can serve as a potential platform for rapid and highly efficient in vitro muscle cell generation.

**Supplementary Information:**

The online version contains supplementary material available at 10.1186/s40580-021-00291-6.

## Introduction

Skeletal muscle is one of the major components in the human body that controls most of the body motions and movements [1, 2]. During the motions, the muscle bundles composed of multiple skeletal muscle cells are contracted or released following the recognition of complex biochemical signals that are mostly generated or transferred from the peripheral and central nervous system through the neuromuscular junctions [3]. Once the muscle tissues are locally damaged, they can be self-healed via multiple sequential steps, including cell division, fusion with the existing muscle fibers, and the final repair [4–6].

However, similar to the regeneration mechanism of other organs, the mass loss of muscle bundles or damages that occur due to genetic abnormalities cannot be restored spontaneously in the absence of external regeneration factors [7, 8]. To address such an issue, in vitro muscle cell generation for transplantation or drug screening purposes has emerged as a promising approach, mostly *via* controlling differentiation of multipotent stem cells or unipotent pre-myogenic cells (i.e., myoblasts) [9–13]. To induce skeletal muscle cell differentiation, one of the most common methods is the use of a cultivation medium containing several myogenic differentiation factors (e.g., FGF, TGF-β, and IGF) [14, 15]. In addition to these soluble factors, it was recently reported that the modulation of the cellular microenvironment, including the extracellular matrix (ECM), can synergistically steer cell fates under the differentiation factor treatments [16–21]. Such strategy includes microgrooving of the cell cultivation platforms that further induces the deformation of cellular shape.

Considering that skeletal muscle tissue retains the elongated structures to give unidirectional forces, modulating cellular morphology into the elongated shape using the microgroove structure is beneficial for efficient muscle cell generation [22]. Various micropatterned structures with different materials, including metals, silicons, proteins, peptides, and biocompatible synthetic polymers, have been reported, all of which are proven to guide cell shape with a high aspect ratio [23–25]. Synthetic polymers (e.g., polydimethylsiloxane, polycaprolactone, polyvinyl alcohol) are particularly advantageous because they are cheap, non-toxic to the cells, and most importantly, can be easily fabricated as various structures via a simple molding technique. However, one of the major drawbacks of such polymers is their hydrophobic property, which prevents cellular adhesion on the platform surfaces [26]. The cell adhesion is extremely important because the majority of cell sources that can generate skeletal muscles in vitro should adhere to the platform surface for further growth and differentiation [27]. Therefore, adding functionality to the polymeric micropatterned substrate is essential for use as the culture substrate for the generation of skeletal muscle cells with improved conversion efficiency [28–32].

**Fig. 1:**
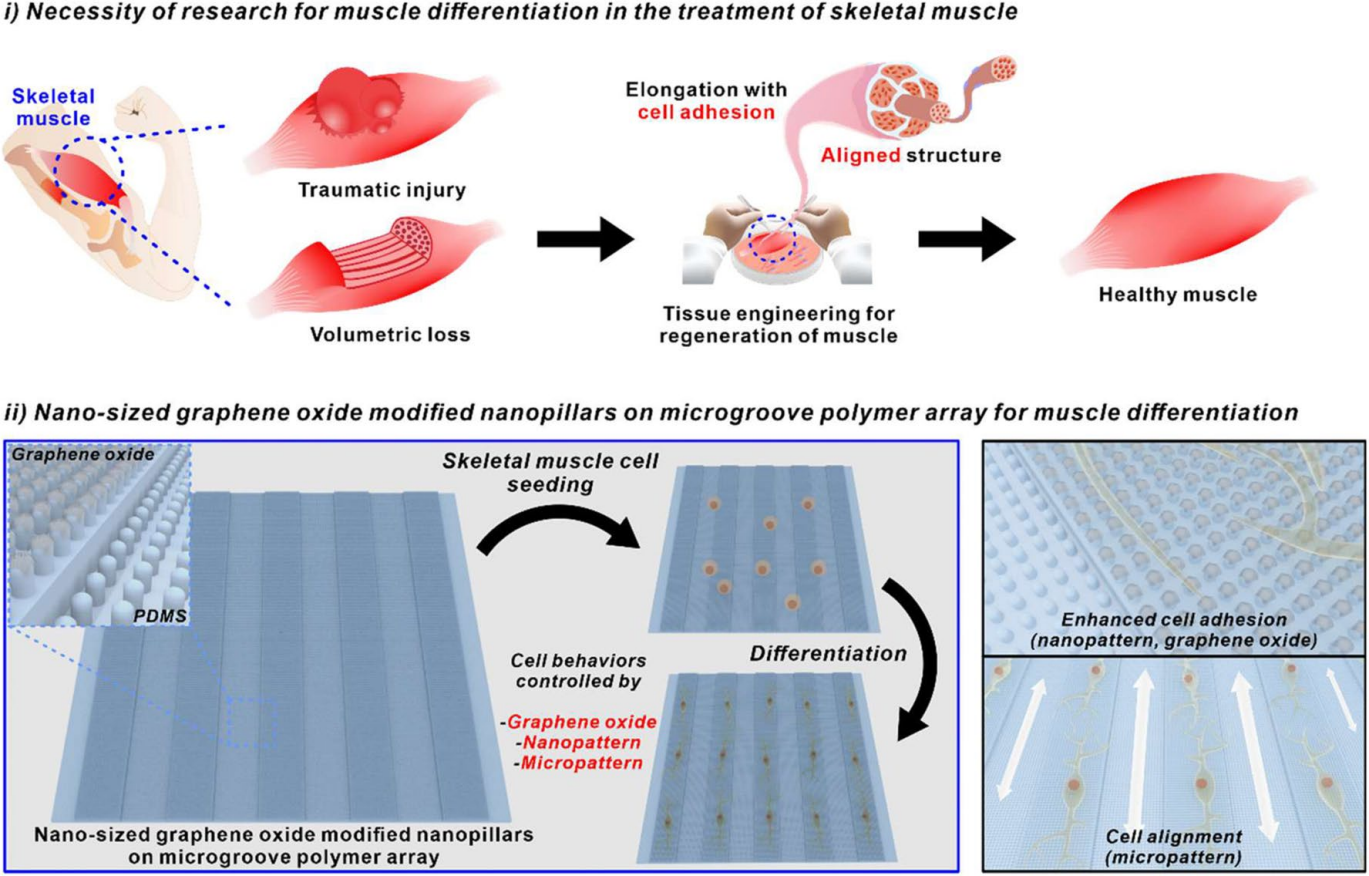
Schematic diagram of necessity of research for muscle differentiation in the treatment of skeletal muscle and nano-sized graphene oxide-modified nanopillars on microgroove polymer array for muscle differentiation

In this study, we report a new platform consisting of nanopillar arrays, nano-sized graphene oxide (sGO), and microgrooves for highly efficient skeletal muscle cell differentiation (Fig. 1). The laser interference lithography technique is first applied to the photoresist (PR)-coated silicon micropattern mold to generate periodic homogeneous nanohole patterns. The polydimethylsiloxane (PDMS) polymer is applied to the nanohole-modified silicon mold to reversely replicate the structure, resulting in the generation of nanopillars on microgroove hybrid polymer array (NMPA). Graphene oxide (GO) of different sizes are applied and coated to the substrates to enhance the cell adhesion on the micropatterned polymeric arrays along with the nanopatterns [33]. The structural and chemical characteristics of GO coated NMPA are confirmed by scanning electron microscopy (SEM) and Raman spectroscopy, respectively. The ability of the fabricated hybrid arrays to generate elongated cell shape is assessed based on three different parameters, including the cell spreading, circularity, and aspect ratio of the myoblast cell line, C2C12. Finally, the efficiency of skeletal muscle cell differentiation of C2C12 on differently fabricated platforms is evaluated based on the immunofluorescence images using myosin heavy chain (MHC) as a myogenesis marker.

## Methods/experimental

### Materials

Distilled water (DW) was purified through a Milli-Q system (Millipore, USA). Sylgard 184 base and curing agent kit were purchased from Dow Corning (Midland, MI, USA) for the fabrication of PDMS substrates. Indium tin oxide (ITO)-coated glass was purchased from Omniscience (Korea). The line-micropatterned silicon wafer of 20 μm in width size was custom-ordered from MicroFIT (Korea). Hexamethyldisilazane (HMDS) was purchased from Sigma-Aldrich (USA), and negative PR (AZ nLOF™ 2020), developer (AZ 300 MIF), and thinner (AZ 1500) were obtained from AZ Electronic Materials (USA). The single-layer GO was purchased from Graphene Supermarket (USA). The quartz plate was purchased from Jinzhou BEST Quartz Glass Co., Ltd. (China). The mouse myoblast cell line (C2C12) was acquired from the American Type Culture Collection (ATCC, USA). To prepare the media, Dulbecco’s modified Eagle’s medium (DMEM) was purchased from Welgene (Korea), and fetal bovine serum (FBS) was purchased from Young In Frontier (Korea). Penicillin, streptomycin, and horse serum were obtained from Gibco (Thermo Fisher Scientific, USA). Before immunostaining the cells, 4% paraformaldehyde from Biosaesang (Korea) was used for fixation. To immunostain the F-actin, Alexa Fluor™ 546 phalloidin was purchased from Thermo Fisher Scientific. Anti-sarcomeric α-actinin antibody (Abcam, UK) and MHC antibody (R&D Systems, USA) were used as primary antibodies. Alexa Fluor™ 488 goat anti-mouse IgG (H+L) (Thermo Fisher Scientific) and goat anti-rabbit IgG (H+L) (Abcam) were used as secondary antibodies.

### Fabrication of nano-, micro-pattern, and NMPA

The micropatterned PDMS of 20 μm in width size was fabricated by casting PDMS in the micropatterned silicon wafer of 20 μm in width size. The nanopatterned PDMS was fabricated by casting PDMS in the nanohole patterns of 400, 600, and 800 nm in size. The nanohole patterns were fabricated using the laser interference lithography technique. In brief, clear ITO substrates were spin-coated with the HMDS and negative PR sequentially. After exposure to the laser, the substrates were baked at 125 °C for 1 min, then immersed in the developer for 1 min. The developed substrates were removed from the developer solution, washed with DW, and dried with N_2_ gas. The NMPA were fabricated by casting PDMS in the silicon wafer patterned with micro−nano holes. The nanohole patterns were fabricated on the micropatterned silicon wafer by the above method. To produce a rigid substrate for cell attachment, PDMS was cast with a 17:1 base-to-curing agent ratio, followed by an overnight cure at 70 °C. The surface of the fabricated patterns was observed by field-emission SEM (Auriga, Carl Zeiss, Germany) after Pt coating by ion-sputter. The topography of the fabricated nanopillars on microgroove was observed by atomic force microscopy (AFM).

### Fabrication of GO thin film and GO modification on NMPA

The fabrication of GO thin film involved the following three processes: (1) GO suspension (50 µL, 500 mg/L in 70% ethanol) was dispensed on a 15 mm^2^ quartz plate. The quartz plate coated with GO suspension was heated to 65 °C for 55 s. (2) The quartz plate heated for 55 s was spin-coated for 65 s at rotation speeds of 500, 1000, and 1800 rpm. Each condition corresponds to the manufacturing conditions of extremely large GO (LGO), 10 nm size of sGO (10-sGO), and 5 nm size of sGO (5-sGO) in sequence. (3) Finally, the GO-coated quartz plate was treated with low-oxygen concentration and low-electrical plasma (LOLP). Limited to LGO manufacturing, LOLP treatment was omitted. During the LOLP treatment, the oxygen concentration was set at 30 sccm, and the electrical power was set at 30 W.

The GO coated NMPA was prepared by contacting a moisturized GO thin film with the NMPA. The GO thin film was exposed for 10 s to steam derived from a 1:1 ratio of a DW**−**DMSO mixture heated at 100 °C for 5 min. The moisturized GO thin film was brought into contact with the NMPA at 25 °C for 180 s. Then, the GO thin film was removed from the NMPA.

### Cell culture and differentiation

The substrates used to culture C2C12 cells were washed with PBS and then treated with UV for 30 min. The cells were incubated in a growth medium containing high-glucose DMEM supplemented with 10% FBS and 1% penicillin/streptomycin for 3 days at 37 °C in a 5% CO_2_ atmosphere. For differentiation, the cells were grown in a differentiation medium containing 2% of horse serum, 1% penicillin/streptomycin, and DMEM. The differentiation medium was replaced every 2 days for 10 days.

### Cell immunostaining

To fix the cells, the substrates were immersed in 4% formaldehyde solution for 10 min. Triton-X solution (0.2%) was used to permeabilize the cells. The cells were blocked using a solution of 1% bovine serum albumin (BSA). Between the steps, the cells were washed three times with Dulbecco’s phosphate-buffered saline (DPBS). After the washing steps, the cells were incubated with a primary antibody (1000:1) at 4 °C overnight. For primary antibodies, sarcomeric α-actinin and MHC were used. The washing steps were repeated three times, and then the cells were incubated with secondary antibodies, Alexa Fluor™ 488 goat anti-mouse IgG (H+L) (FITC) and goat anti-rabbit IgG (H+L), for 2 h at room temperature. Cell nuclei were stained with Hoechst (3 µg/mL) for 3 min, and F-actin was stained with Alexa Fluor™ 546 phalloidin. Immunostained cells were observed using a confocal laser scanning microscope (Carl Zeiss GmbH, Jena, Germany). Confocal images were analyzed using ZEN Black software (Carl Zeiss GmbH).

## Results and discussion

### Cell behaviors on nano- and micropatterns

**Fig. 2:**
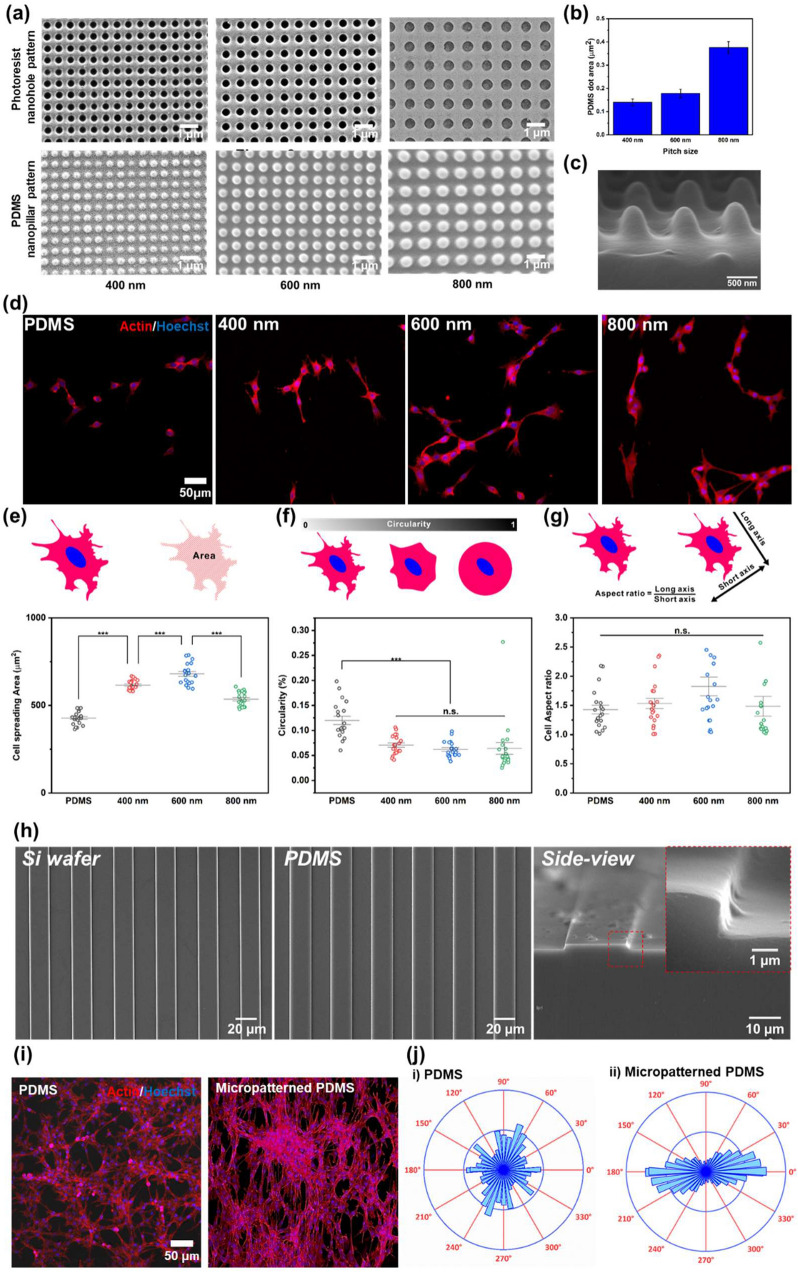
Confirmation of the effect of nano- and micropatterns for cell behaviors. **a** FE-SEM images of PR nanohole patterns and PDMS nanopillar patterns, and **b** size distribution of PDMS patterns. **c** 3D SEM image of PDMS nanopillar patterns. **d** Confocal microscope images of the cells on bare PDMS, and 400, 600, and 800 nm PDMS patterns (left to right) immunostained with actin (Red) and Hoechst (Blue). **e**−**g** Cell spreading area (**e**), circularity (**f**), and cell aspect ratio (**g**) of the cells on bare PDMS, and 400, 600, and 800 nm PDMS pattern. **h** FE-SEM images of micropatterned silicon wafer, PDMS and side view image of micropatterned PDMS. **i** Confocal microscope images of the cells on bare PDMS and micropatterned PDMS immunostained with actin (Red) and Hoechst (Blue). **j** Cell orientation analysis of the cells on the bare PDMS and micropatterned PDMS. (*** *p* ≤ 0.001)

To confirm the ability of nanopillars to increase cell adhesion on generated on PDMS substrates, C2C12, an immortalized mouse myoblast, was chosen as a model cell line [34]. C2C12 is an adherent cell line and should be cultivated on a surface that is functionalized to promote cell adhesion. Interestingly, periodic nanostructures, including nanogrooves and nanopillars, have proven effective in enhancing cell adhesion even without the use of ECM proteins and peptides [35–38]. Therefore, in this study, the uniform PDMS nanopillar patterns were fabricated by laser interference lithography, as specified in Additional file 1: Fig. S1 [23]. To investigate the effects of nanopattern sizes on cell adhesion and morphology, nanohole with different sizes (e.g., 400, 600, and 800 nm) were generated on the silicon mold and were reversely replicated using PDMS, as shown in Fig. 2a. Based on the size distribution result (Fig. 2b) and high-resolution vertical SEM image (Fig. 2c), we found that all the PDMS nanopillars were uniformly fabricated. Such uniformity in size and shape is reported to be critical for facilitating cell adhesion to the desired substrates [23].

As shown in the images of the cells double-stained for F-actin and nuclei (Fig. 2d), C2C12 cells on the nanopatterned PDMS showed a more elongated and stretched morphology than those on bare PDMS, regardless of the nanopattern sizes. For intensive analysis of cell behavior on each substrate, we calculated the cell spreading area, circularity, and cell aspect ratio based on multiple actin/DAPI fluorescence cells (Fig. 2e−g). The cell spreading area on nanopatterned PDMS was increased compared with the bare PDMS substrate (Fig. 2e). Specifically, the 600 nm-sized nanopatterned PDMS showed a cell spreading area of 679.7 µm^2^, which was significantly greater than those of the other substrates (428.2 µm^2^ for bare PDMS, 616.3 µm^2^ for 400 nm-sized nanopatterned PDMS, and 535.0 µm^2^ for 800 nm-sized nanopatterned PDMS).

The cell circularity was considered next. Cells with a circularity of 1 are perfectly circular without any stretch from the surface and structural deformation. By contrast, a circularity of 0 means that the roughness of the outer area of the cell is extremely high, which is considered as cell growth. We found that the highest circularity corresponded to bare PDMS, showing a value of 0.12, followed by the 400-, 600-, and 800 nm-sized nanopatterned substrates, with values of 0.071, 0.062, and 0.064, respectively (Fig. 2f). Similar to the cell spreading results, the 600 nm-sized nanopatterned substrate showed the lowest circularity among all the groups, indicating that more branches stretch out from the cells; that is, the cells show enhanced adhesion. Unlike these two parameters, the cell aspect ratio was not affected by the nanopatterns, as shown in Fig. 2g. Taken together, we found that the 600 nm-sized PDMS nanopillar arrays are highly effective in enhancing cell adhesion and spreading.

In addition to an effort to improve cell attachment to the platform, we further investigated the effects of PDMS microgroove patterns on cell morphology. The fabrication process of the micropattern is described in Additional file 1: Fig. S2. As shown in Fig. 2h, the micropatterns on the fabricated micropatterned PDMS were uniformly aligned in one direction. The size and height of the micropatterns on the PDMS were about 20 and 2 μm, respectively, which is consistent with those of the micropatterns on the silicon wafers. After the C2C12 cells were seeded on the micropatterned PDMS, the cells were immunostained to confirm the alignment of the cells (Fig. 2i). As shown in the confocal microscopy images, aligned microstructures of the C2C12 cells were obtained on the micropatterned PDMS compared with the cells on the bare PDMS. To acquire the quantitative data on the cell alignment from the images, spatial point pattern analysis was undertaken using the J-function in the ImageJ program. An initial color survey of the cells on the substrates was performed (Additional file 1: Fig. S3). It revealed that the cells on the bare PDMS showed randomly arranged structures, and the bar graph demonstrated the absence of any dominant angle of cell alignment (Fig. 2j). By contrast, the cells on the micropatterned PDMS were aligned horizontally, and 73.6% of the intensity was focused within 30° from a dominant line that represented 0° in the graph. Therefore, we could adapt the micropatterned PDMS to fabricate the NMPA for cell alignment.

### Cell behavior on various GO-modified substrates

**Fig. 3:**
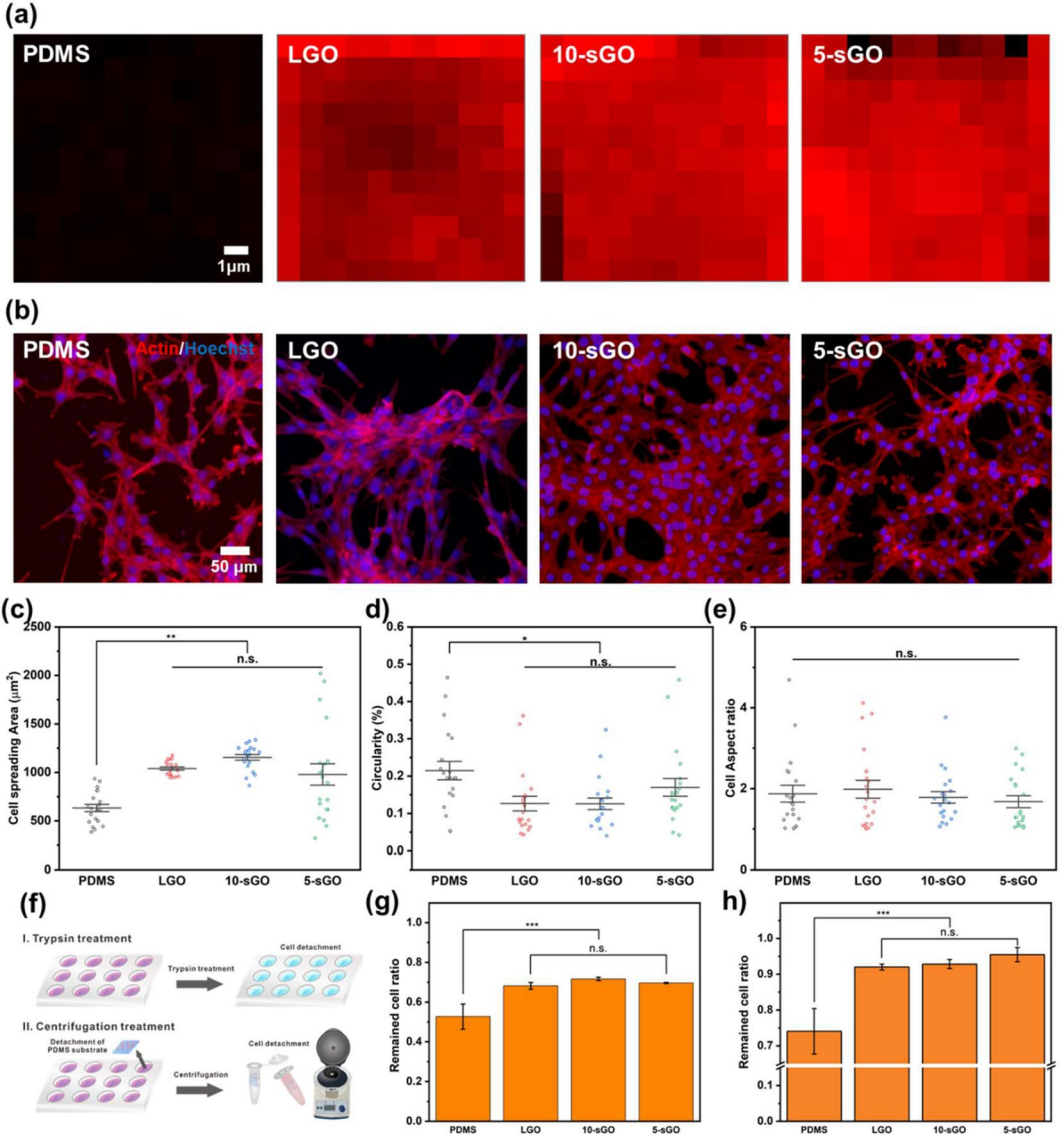
Confirmation of the effect of graphene oxide on cell behaviors. **a** FE-SEM images of PR nanohole patterns and PDMS nanopillar patterns, and **b** size distribution of PDMS patterns. Raman intensity map of the bare PDMS, LGO-, 10-sGO-, and 5-sGO-modified PDMS. **b** Confocal microscope images of the cells on bare PDMS, LGO-, 10-sGO-, and 5-sGO-modified PDMS immunostained with actin (Red) and Hoechst (Blue). **c−e** Cell spreading area (**c**), circularity (**d**), and cell aspect ratio (**e**) of the cells on bare PDMS, LGO-, 10-sGO-, and 5-sGO-modified PDMS. **f** Schematic diagram of trypsin and centrifugation treatment process. **g**, **h** Cell ratio remaining on the PDMS, LGO-, 10-sGO-, and 5-sGO-modified PDMS after trypsin (**g**) and centrifugation (**h**) treatment. (* *p* ≤ 0.5, ** *p* ≤ 0.01, *** *p* ≤ 0.001)

GO have been used as composing materials for various bio-integrated platforms due to biocompatible characteristics and highly adhesive nature of GO [39, 40]. To attach the C2C12 cells to the substrates stably and further control the myogenesis, GO sheets were coated on the substrates through a transfer technique, as previously reported [41]. The GO could be simply modified on the desired substrate via the drop-coating method. However, the large stacked GO sheets would cover the entire polymer surface and could thus eliminate the nanopattern effects. Therefore, three different types of GO, including LGO, 10-sGO, and 5-sGO, were applied to the substrates. The transfer of the GO onto the PDMS substrates is described in Additional file 1: Fig. S4. As shown in Fig. 3a and Additional file 1: Fig. S5, all types of GO were successfully transferred to the PDMS substrates regardless of their size. As a proven cytophilic material, the spreading area of C2C12 cells was increased on all GO-coated substrates (Fig. 3b and c). Specifically, the cell spreading area was calculated to be 633.1, 978.3, 1039.5, and 1154.6 µm^2^ for bare PDMS, 5-sGO, LGO, and 10-sGO substrates, respectively. Moreover, the circularity values were 0.13, 0.13, and 0.17 for the LGO, 10-sGO, and 5-sGO groups, respectively (Fig. 3d), indicating that GO induces branch-like formations that stretch out from the outer cell membrane area due, in part, to the enhanced filopodia and lamellipodia formation. No significant differences were observed in the cell aspect ratios among all tested groups, proving the excellent cytophilic property of GO that is similar to the periodic nanopatterns.

To further study the cell adhesion strength on GO coated NMPA, the cell adhesion was intentionally weakened via trypsin treatment and centrifugation as chemical and mechanical methods, respectively (Fig. 3f). The trypsin treatment time and rotating speed (i.e., centrifugal force) were optimized as 80 s and 8 min, respectively (Additional file 1: Fig. S6). The centrifugal force applied to the cells was calculated to be 0.269 mN based on the following equation:

1 $$\text{F}=m\times {\omega }^{2}r$$

After 80 s of the trypsin treatment, 52.7% of the cells remained on the bare PDMS substrate, while 68.2%, 71.7%, and 69.6% of the cells remained attached to the LGO-, 10-sGO-, and 5-sGO-coated PDMS substrates, respectively (Fig. 3g). In the mechanical detachment experiments, 74.0%, 92.0%, 92.8%, and 95.5% of the cells were found to be attached to the bare PDMS, LGO-, 10-sGO-, and 5-sGO-coated PDMS substrates, respectively, when the centrifugal force was applied for 8 min (Fig. 3h). Taken together, the results indicate that GO is effective in not only enhancing the cell spreading (i.e., cell adhesion area) but also the cell adhesion on the artificial polymer substrates, both of which are incredibly important for *in vitro* skeletal muscle cell differentiation.

### Cell behaviors on GO coated NMPA

**Fig. 4:**
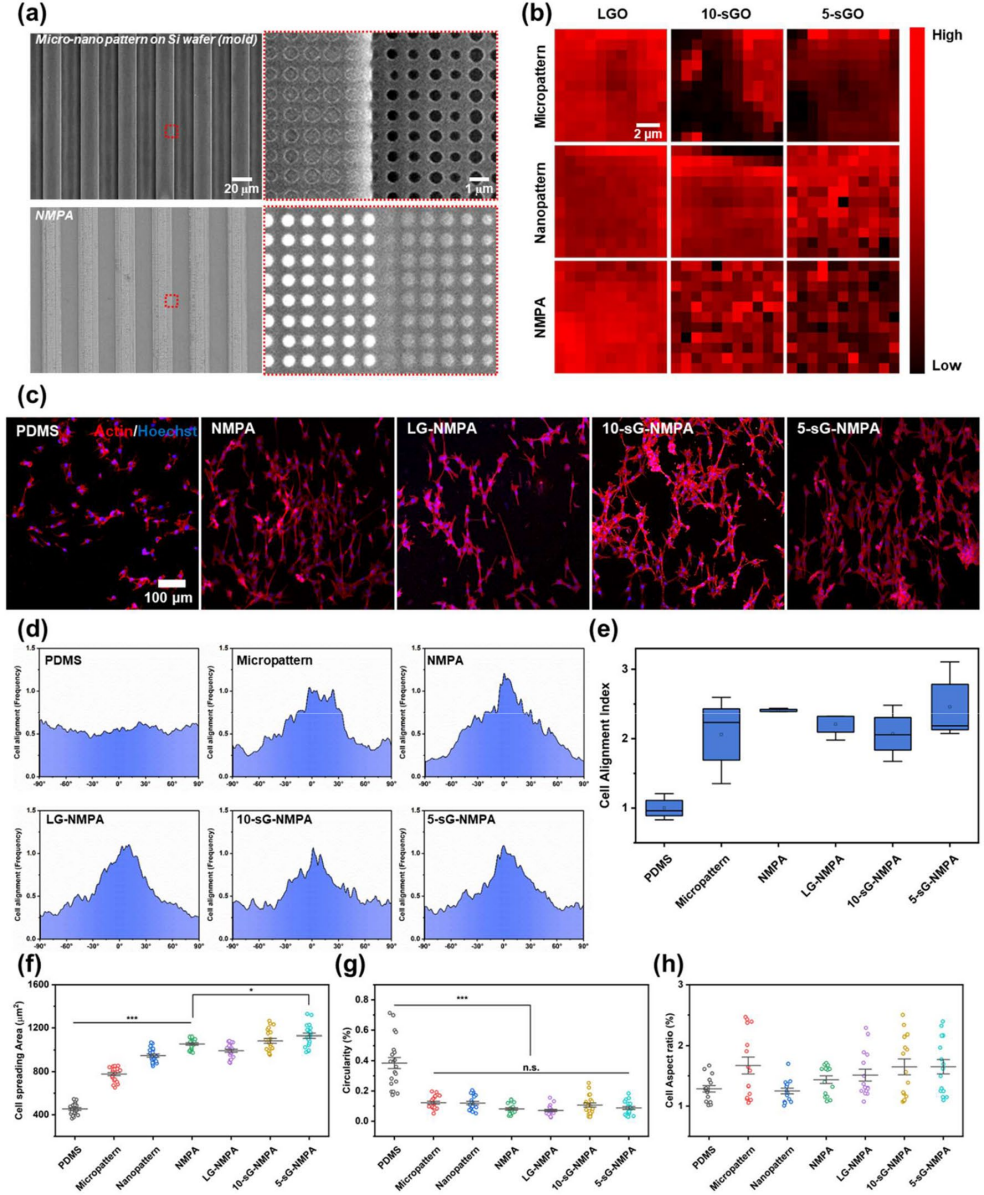
Investigation of the cell behaviors on the GO-coated NMPA. **a** FE-SEM images of micro-nano pattern on silicon wafer and NMPA. **b** Raman intensity map of the LGO-, 10-sGO-, and 5-sGO-coated micropattern, nanopattern, and NMPA. **c** Confocal microscope images of the cells on bare PDMS, NMPA, LG-NMPA, 10-sG-NMPA, and 5-sGO-NMPA immunostained with actin (Red) and Hoechst (Blue). **d** The cell orientation graph of the cells on the bare PDMS, micropatterned PDMS, NMPA, LG-NMPA, 10-sG-NMPA, and 5-sGO-NMPA. **e** Cell alignment index of the cells on bare PDMS, micropatterned PDMS, NMPA, LG-NMPA, 10-sG-NMPA, and 5-sGO-NMPA. **f**−**h** Cell spreading area (**f**), circularity (**g**), and cell aspect ratio (**h**) of the cells on the bare PDMS, micropatterned PDMS, nanopatterned PDMS, NMPA, LG-NMPA, 10-sG-NMPA, and 5-sGO-NMPA. (* *p* ≤ 0.5, *** *p* ≤ 0.001)

After confirming the excellent properties of each core component, including PDMS nanopillars and sGO for improving cell adhesion, and the microgrooves for cell morphology modulation, we attempted to fabricate all combined sG-NMPA (Additional file 1: Fig. S7). As shown in Fig. 4a, the 600 nm-sized nanohole patterns were successfully fabricated on the 20 μm-sized microgrooved Si wafer. Then, PDMS was used to reversely replicate the structure of the nanohole pattern-modified Si wafer. The height of fabricated nanopillars on microgroove was 121.98 nm (Additional file 1: Fig. S8). Afterward, LGO, 10-sGO, and 5-sGO were modified on each nano-, micro-patterned PDMS, and NMPA substrate via the contact-printing method under high humidity. As shown in Fig. 4b, owing to the size of LGO (average size: 0.5**−**10 μm), they were heavily stacked on the surface, resulting in uniform G-band (i.e., in-plane vibration of sp^2^-bonded carbon atoms) distribution in the Raman spectrum for all the substrates regardless of their topographical differences. For 10-sGO and 5-sGO, the structural morphology of both microgrooves and nanopillars resulted in variations of G-band intensity (Fig. 4b). Among microgrooves and nanopatterns, the PDMS nanopillars showed superior GO absorption efficiency over the microgrooves-only substrate due to increased hydrophobic property, which lowered the surface energy level. After confirming successful GO transfer to NMPA, C2C12 cells were seeded on each platform and stained with phalloidin and Hoechst to visualize F-actin expression and the nucleus shape, respectively (Fig. 4c). We specifically focused on the alignment and direction of the C2C12 cells, which are important indicators to guide skeletal muscle cell differentiation through cell morphology manipulation. As hypothesized, cells on the bare PDMS substrate showed random orientation and growth with limited cell size. However, the patterned PDMS substrates forced cells to exhibit a unidirectional morphology with a close cell-to-cell network that mimics the structure of in vivo muscle tissue (Fig. 4d). To better compare the cell alignment tendency on each substrate, the degree of alignment was converted to numerical values based on the following equation: [42], when σ_cell_ is the standard deviation of cell angles.

2 $${\text{Cell alignment index = }}\frac{{\left( {180/\sqrt {12} } \right)}}{{{\sigma _{{\text{cell}}}}}}$$

As shown in Fig. 4e, the calculated cell alignment index of cells on patterned substrates was 2.06, 2.41, 2.21, 2.07, and 2.50 for microgrooves, NMPA, LGO-coated NMPA (LG-NMPA), 10-sGO-coated NMPA (10-sG-NMPA), and 5-sGO-coated NMPA (5-sG-NMPA), respectively. The results indicate that microgrooves are critical in guiding cellular morphology into an elongated shape, while sGO modification does not harm the guiding ability of microgrooves, especially 5-sGO. Regarding the effects of GO modification, cell spreading increased on the bare PDMS nanopattern and the GO-coated substrates, while the circularity decreased for cells from all the substrates compared with bare PDMS, which is consistent with the results we obtained without microgrooves (Figs. 2e−g and 3c−e). Taken together, we can conclude that the developed sG-NMPA are highly effective in both manipulating cell morphology into an elongated shape and enhancing cell adhesion, which is advantageous for the generation of skeletal muscle cell differentiation.

### Enhanced skeletal muscle cell differentiation on GO-coated NMPA

**Fig. 5:**
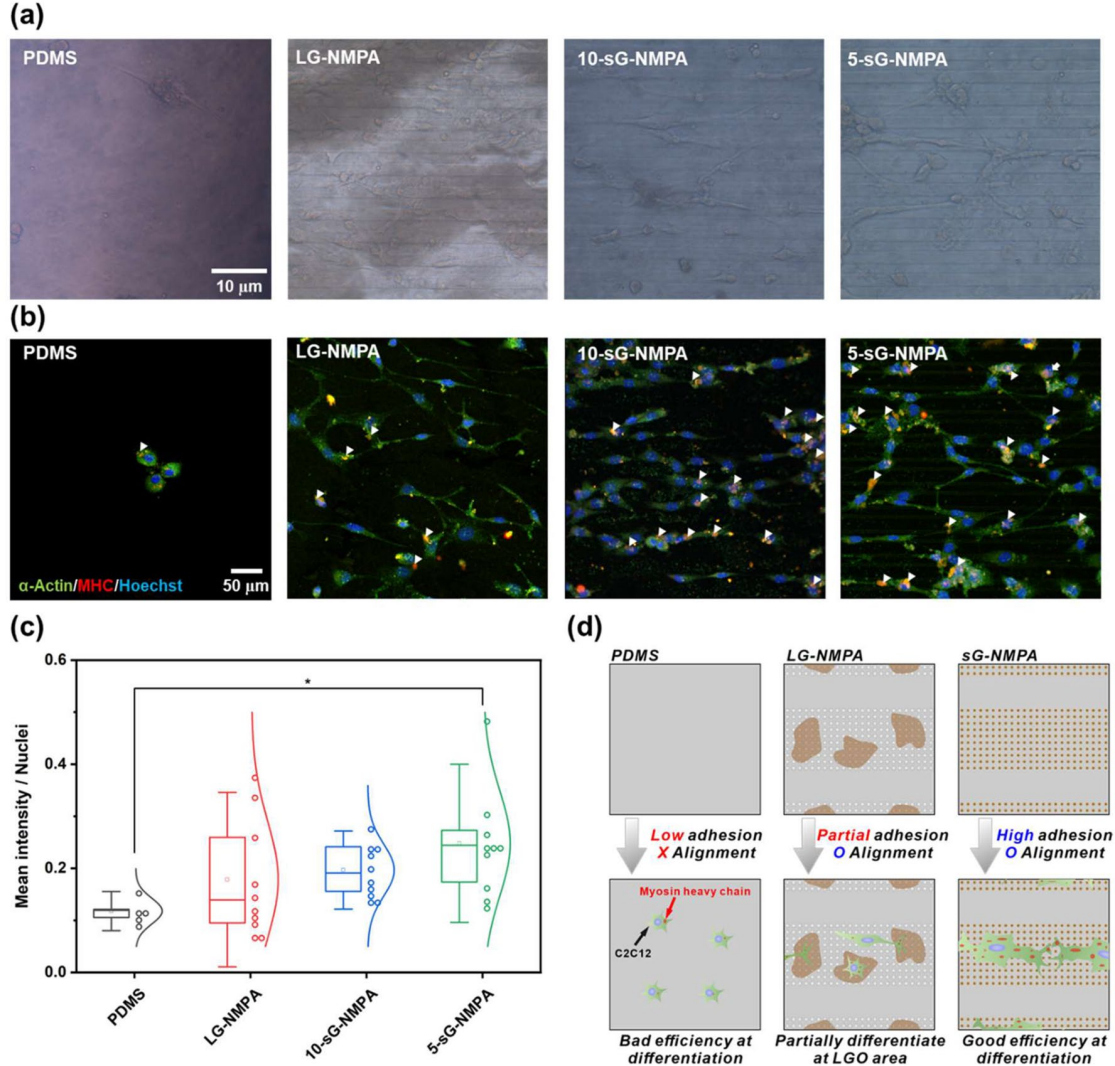
Confirmation of myogenic differentiation of the cells. **a** Brightfield images of the cells on the bare PDMS, LG-NMPA, 10-sG-NMPA, and 5-sGO-NMPA. **b** Confocal microscope images of the cells on bare PDMS, LG-NMPA, 10-sG-NMPA, and 5-sGO-NMPA immunostained with α-actin (Green), MHC (Red), and Hoechst (Blue). **c** The graph for mean intensity divided by number of nuclei. **d** Schematic diagram of the correlation between GO size and cell differentiation based on the mean intensity data. (* *p* ≤ 0.5)

To confirm the ability of the fabricated hybrid platform to guide myogenic differentiation, C2C12 cells were finally cultured. As hypothesized, cells on bare PDMS were detached immediately due to the low cell adhesion strength (Additional file 1: Fig. S9, Additional file 2: Video S1). The detachment of C2C12 cells on flat PDMS surfaces usually occurs owing to the high contractility of the cells compared to their adhesion strength to the substrates [43, 44]. Remarkably, by contrast, cells on the GO-coated nanopillar-modified microgrooves were observed to be stable throughout the differentiation. After 10 days of differentiation, cells remaining on PDMS surfaces showed a circular shape, having a low morphological aspect ratio and high circularity. However, the cells on the GO-coated NMPA substrates showed higher cell spreading area and more elongated morphology than the cells on the bare PDMS. After that, the differentiated cells were further stained with two different myogenesis markers, α-actinin and MHC (Fig. 5b). α-Actinin is an actin-binding cytoskeletal protein critical for focal adhesion [45]. MHC is one of the motor proteins in muscle filaments [46]. As shown in Fig. 5b, both α-actinin and MHC were highly expressed in the GO-modified nanopillar groups, especially in sGO-coated substrates. To better compare the skeletal differentiation based on the immunofluorescence images, the mean intensities of MHC expressed as red color were divided by the number of nuclei and plotted. The mean values of the differentiated cells were calculated to be 0.118, 0.178, 0.197, and 0.248 for bare PDMS, LG-NMPA, 10-sG-NMPA, and 5-sG-NMPA, respectively (Fig. 5c). Although the variations were found to be high for all the groups, sG-NMPA showed 10.7% (5 nm) and 39.3% (10 nm) higher MHC expression levels than the LGO-coated nanopillar-modified microgrooves. Based on this observation, we concluded that sGO conserves the distinct nanopillar and microgroove co-existing structure of the fabricated PDMS substrate and also enhances cell adhesion, which results in enhanced skeletal muscle cell differentiation (Fig. 5d).

## Conclusions

In this study, a GO-coated NMPA that enhances skeletal muscle cell differentiation was fabricated. The micropattern, nanopattern, and GO were employed in the hybrid pattern array for guiding cell differentiation and function. Firstly, the micropattern was used for cell alignment with the pattern. Next, the nanopattern and GO were used to enhance the adhesion of cells on the polymer substrate. Altogether, the hybrid pattern array showed enhanced cell spreading area, including cell alignment. Similar to the cell behaviors on the hybrid pattern array, the myogenic differentiation of the cells on the hybrid pattern array was enhanced compared with the bare PDMS substrate. Furthermore, we investigated the optimized condition of the GO, especially the size of the GO on the patterned substrate. From the results, 5 nm-sized GO on the hybrid pattern array enhanced the differentiation of cells and stable culture of the cells on the polymer substrate simultaneously. The results indicate that the proposed GO-coated NMPA is an appropriate platform for skeletal muscle cell differentiation on the rigid polymer substrate. Therefore, the sGO-coated NMPA can be utilized in regenerative medicine, which requires control of cell differentiation and is a fundamental technology of biorobots composed of muscle cells and the polymer substrate.

## Supplementary Information

**Additional file 1: Fig. S1.** Schematic diagram of the fabrication process of PDMS nanopattern. **Fig. S2.** Schematic diagram of the fabrication process of PDMS micropattern. **Fig. S3.** Color survey data of aligned cells on the bare PDMS (a) and micropatterned PDMS (b). **Fig. S4.** Schematic diagram of the fabrication process of GO-coated PDMS substrates. **Fig. S5.** Raman spectrum of the bare PDMS, and LGO-, 10-sGO, and 5-sGO-coated PDMS substrates. **Fig. S6.**. Optimization of trypsin (a) and centrifugation (b) treatment time for the cells on bare PDMS substrate. (* p ≤ 0.5, ** p ≤ 0.01, *** p ≤ 0.001). **Fig. S7.** Schematic diagram of the fabrication process of GO-coated NMPA. **Fig. S8.** Topology analysis of nanopillars on microgroove. AFM image (a) and height size and pitch size (b) of nanopillars on microgroove. **Fig. S9.** Morphology of the cells on the bare PDMS (a), NMPA (b), and 5-sG-NMPA (c) after 5 days of differentiation.

**Additional file 2: Video S1.** Detachment of the skeletal muscle cells from the bare PDMS substrate. Video plays at 8x speed.

## Data Availability

The datasets used and/or analyzed during the current study are available from the corresponding author on reasonable request.
