# Supplementary material for: Nano-sized graphene oxide coated nanopillars on microgroove polymer arrays that enhance skeletal muscle cell differentiation
Source: Nano Converg. 2021 Dec 4;8:40. doi: 10.1186/s40580-021-00291-6 (PMC8643291; doi:10.1186/s40580-021-00291-6)
Supplement: Supplementary file 1 — Additional file 1: Fig. S1. Schematic diagram of the fabrication process of PDMS nanopattern. Fig. S2. Schematic diagram of the fabrication process of PDMS micropattern. Fig. S3. Color survey data of aligned cells on the bare PDMS (a) and micropatterned PDMS (b). Fig. S4. Schematic diagram of the fabrication process of GO-coated PDMS substrates. Fig. S5. Raman spectrum of the bare PDMS, and LGO-, 10-sGO, and 5-sGO-coated PDMS substrates. Fig. S6.. Optimization of trypsin (a) and centrifugation (b) treatment time for the cells on bare PDMS substrate. (* p ≤ 0.5, ** p ≤ 0.01, *** p ≤ 0.001). Fig. S7. Schematic diagram of the fabrication process of GO-coated NMPA. Fig. S8. Topology analysis of nanopillars on microgroove. AFM image (a) and height size and pitch size (b) of nanopillars on microgroove. Fig. S9. Morphology of the cells on the bare PDMS (a), NMPA (b), and 5-sG-NMPA (c) after 5 days of differentiation. [file 40580_2021_291_MOESM1_ESM.doc]

Additional file 1

**Nano-sized graphene oxide coated nanopillars on microgroove polymer arrays that enhance skeletal muscle cell differentiation**

Hye Kyu Choi^1,†^, Cheol-Hwi Kim^2,†^, Sang Nam Lee^3,*^, Tae-Hyung Kim^2,*^, and Byung-Keun Oh^1,*^

^1^ Department of Chemical & Biomolecular Engineering, Sogang University, Seoul 04170, South Korea

^2^ School of Integrative Engineering Chung-Ang University, Seoul 06974, Korea

^3^Uniance Gene Inc., Seoul 04107, South Korea

^†^These authors contributed equally to this work

*Correspondence: Byung-Keun Oh, Tae-Hyung Kim, Sang Nam Lee

Tel.: +82-2-820-5469

E-mail address: bkoh@sogang.ac.kr, thkim0512@cau.ac.kr


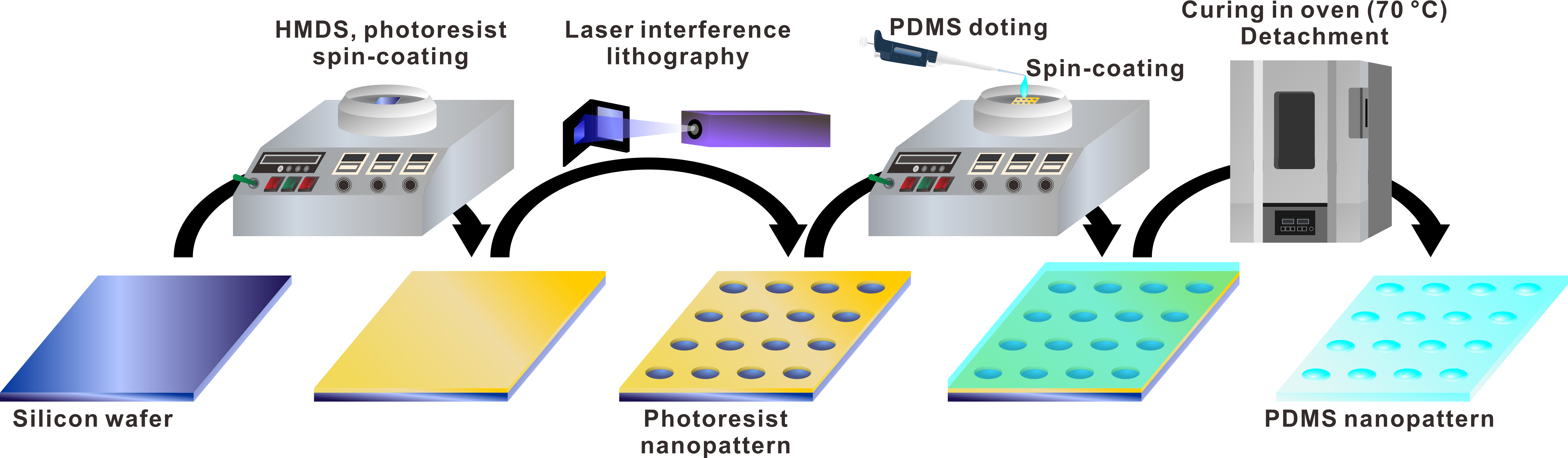


**Figure S1.** Schematic diagram of the fabrication process of PDMS nanopattern.


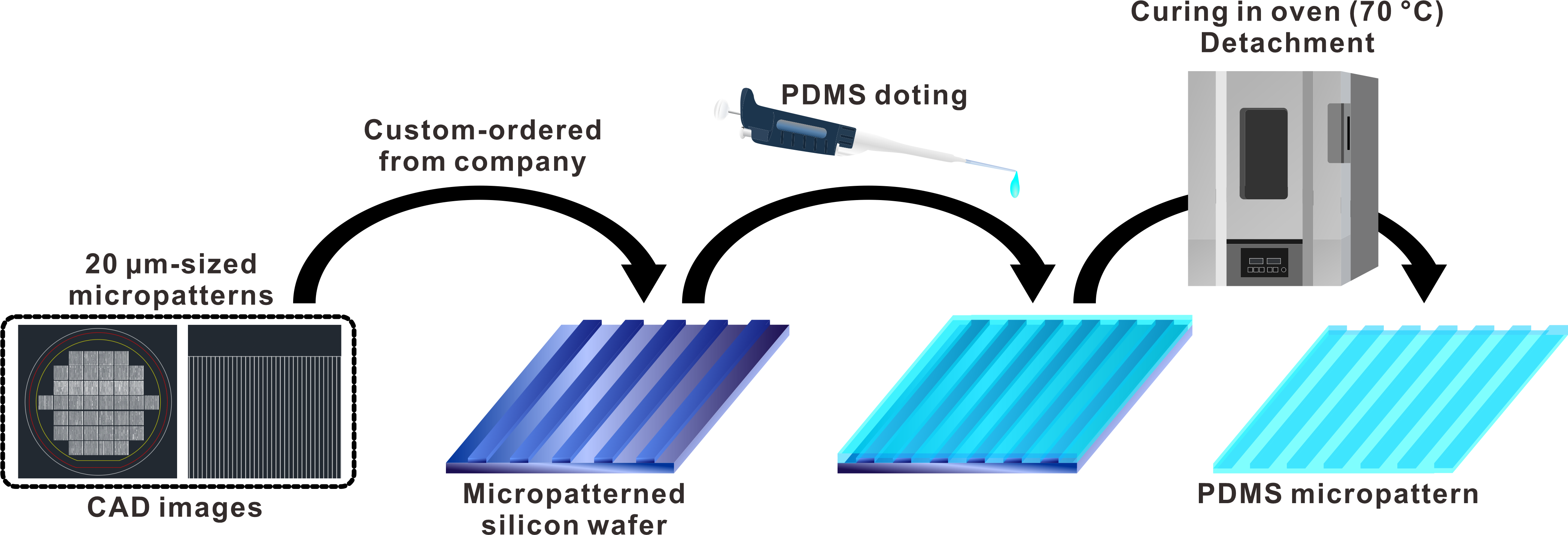


**Figure S2.** Schematic diagram of the fabrication process of PDMS micropattern.


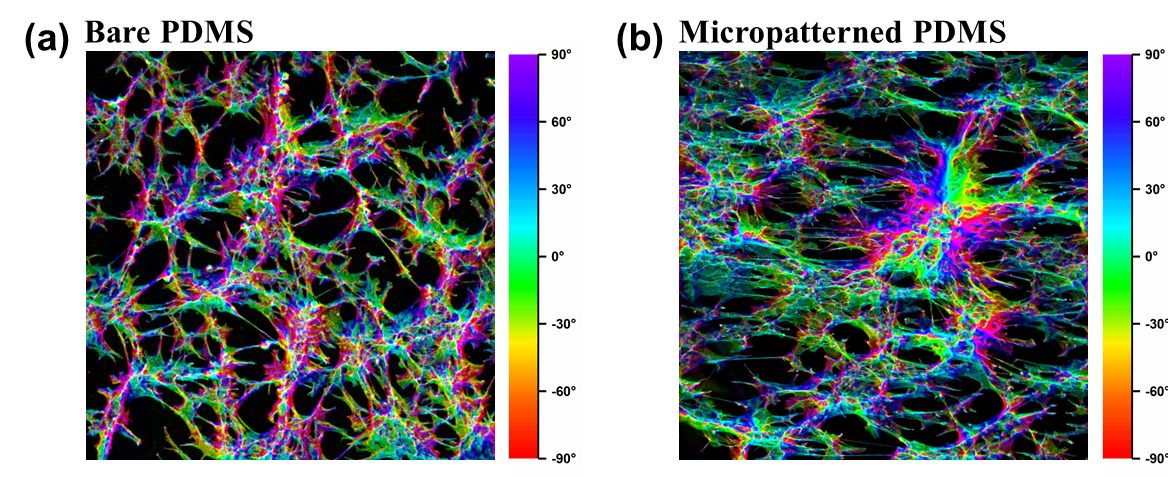


**Figure S3.** Color survey data of aligned cells on the bare PDMS (**a**) and micropatterned PDMS (**b**).


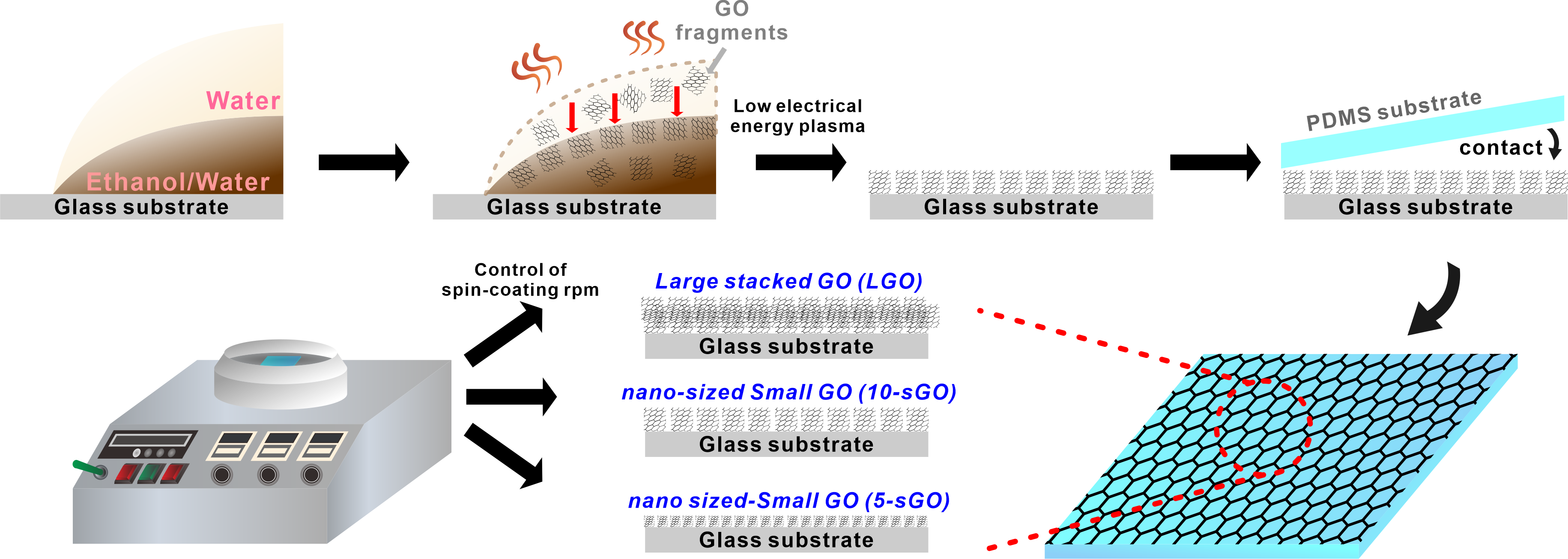


**Figure S4.** Schematic diagram of the fabrication process of GO-coated PDMS substrates.


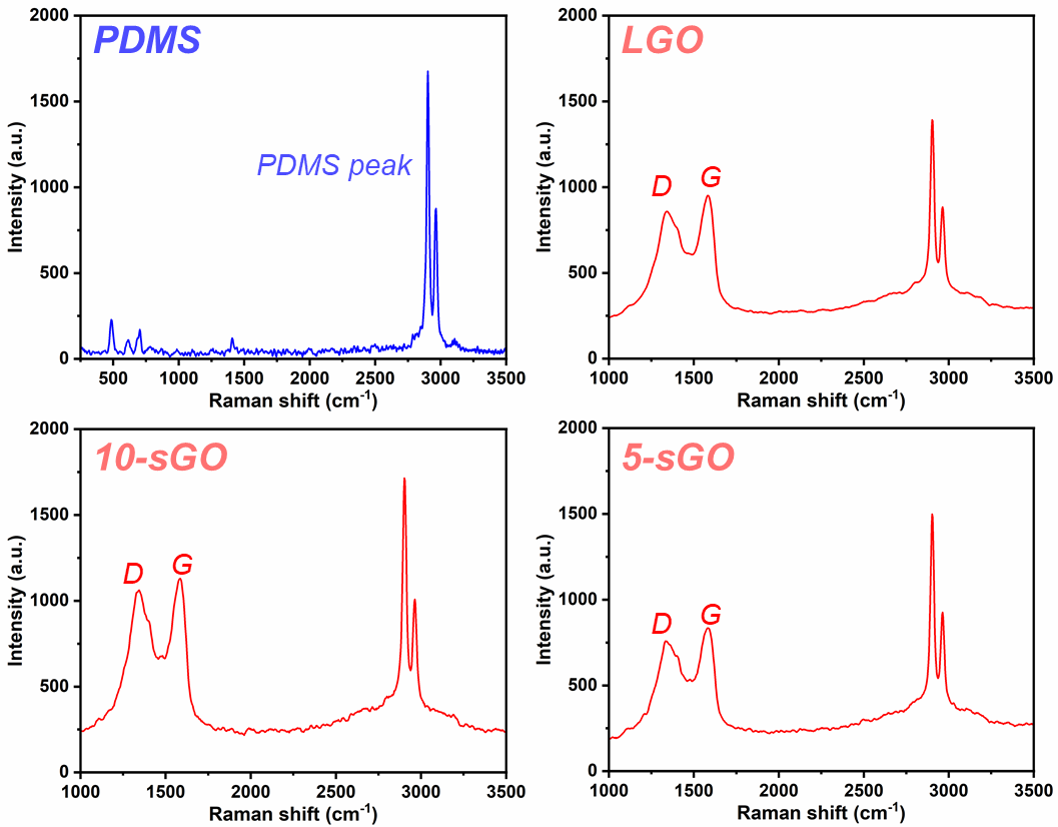


**Figure S5.** Raman spectrum of the bare PDMS, and LGO-, 10-sGO, and 5-sGO-coated PDMS substrates.


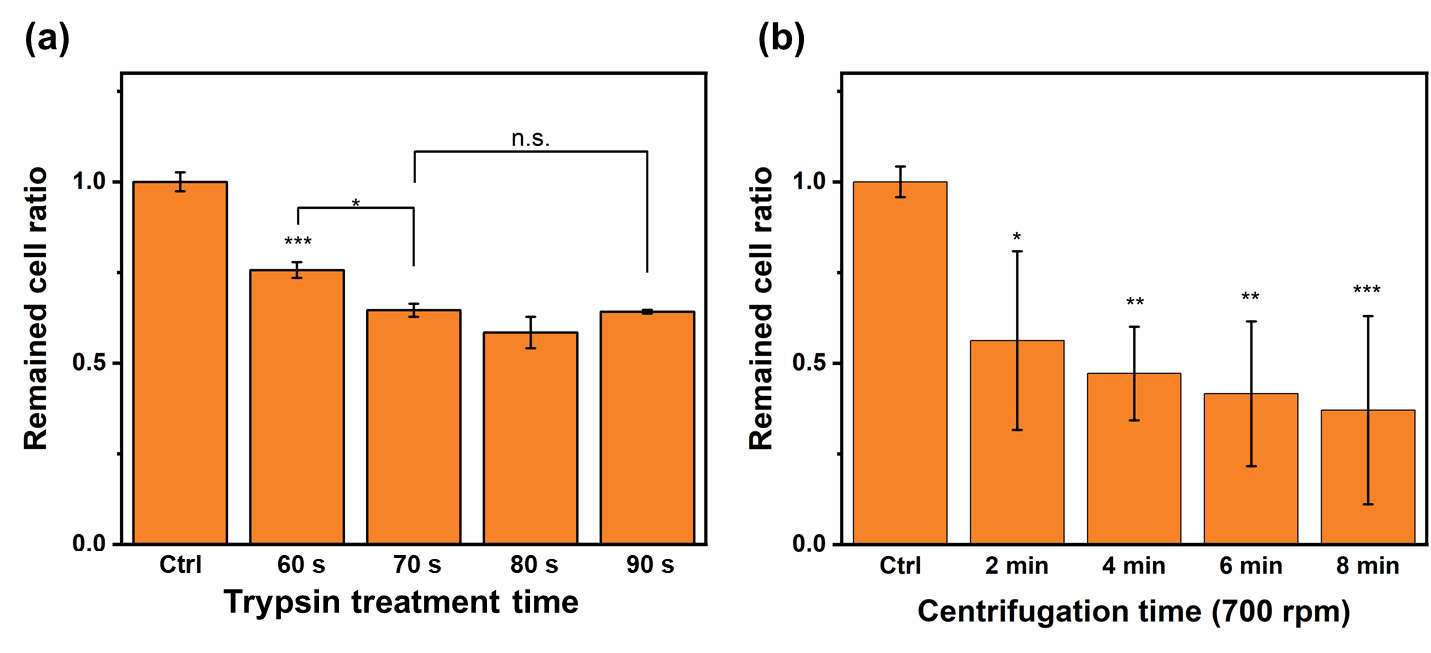


**Figure S6.** Optimization of trypsin (**a**) and centrifugation (**b**) treatment time for the cells on bare PDMS substrate. (* *p* ≤ 0.5, ** *p* ≤ 0.01, *** *p* ≤ 0.001)


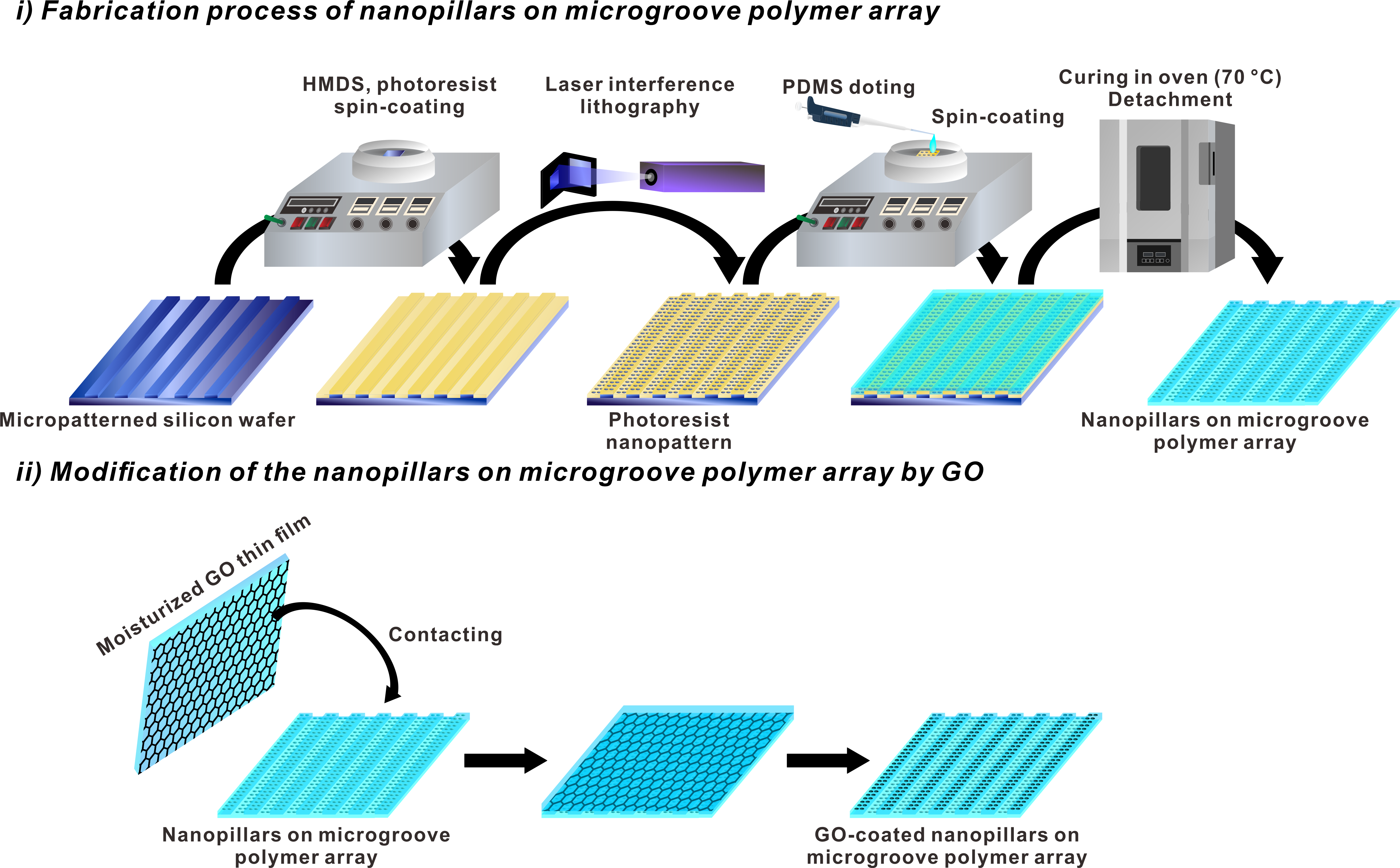


**Figure S7.** Schematic diagram of the fabrication process of GO-coated NMPA.


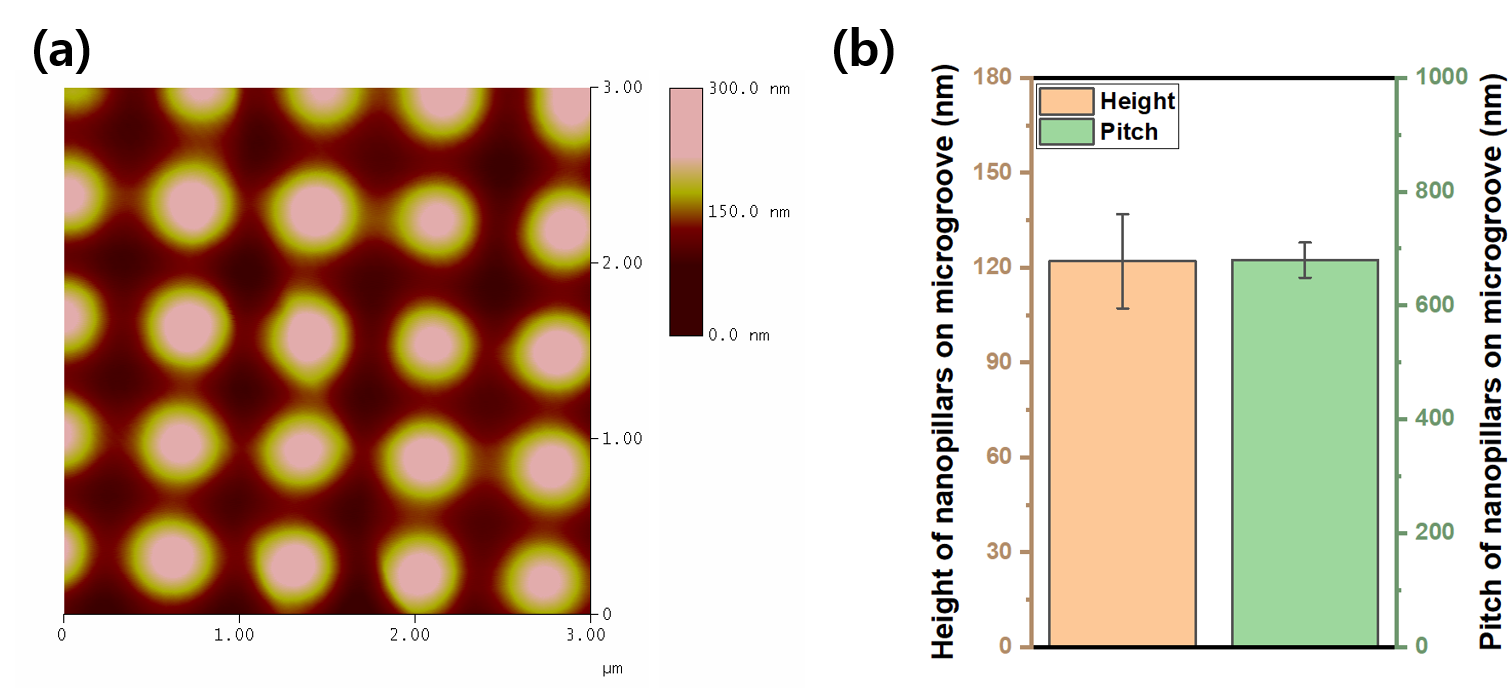


**Figure S8.** Topology analysis of nanopillars on microgroove. AFM image (a) and height size and pitch size (b) of nanopillars on microgroove.


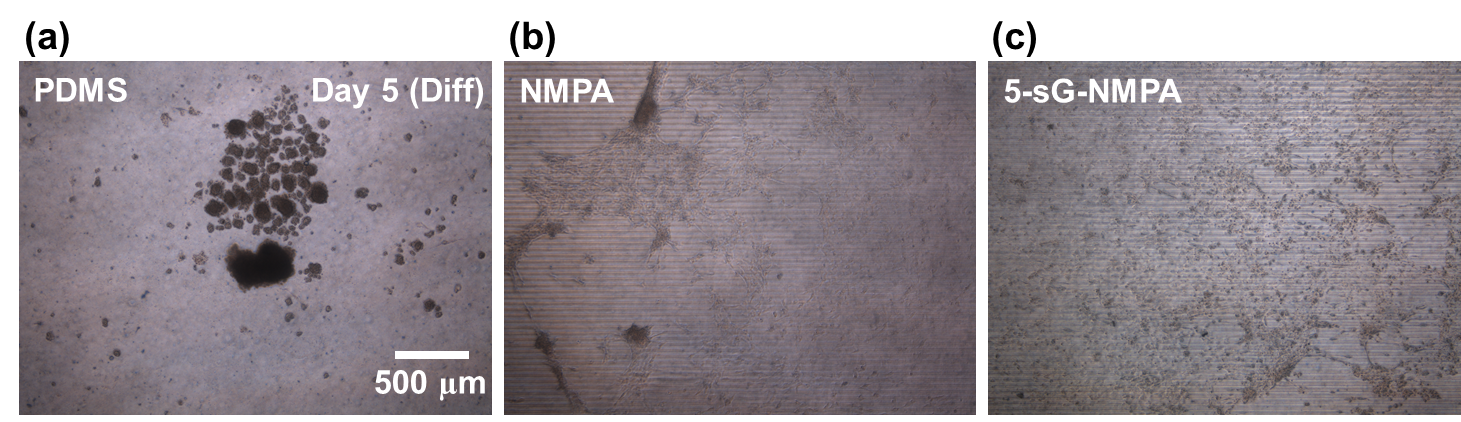


**Figure S9.** Morphology of the cells on the bare PDMS **(a)**, NMPA **(b)**, and 5-sG-NMPA **(c)** after 5 days of differentiation.
